# Effects of exergames on heart rate variability of women with fibromyalgia: A randomized controlled trial

**DOI:** 10.1038/s41598-020-61617-8

**Published:** 2020-03-20

**Authors:** Santos Villafaina, Daniel Collado-Mateo, Francisco J. Domínguez-Muñoz, Narcis Gusi, Juan P. Fuentes-Garcia

**Affiliations:** 1 Physical Activity and Quality of Life Research Group (AFYCAV). Faculty of Sport Science, University of Extremadura, Extremadura, Spain; 2 Centre for Sport Studies, Rey Juan Carlos University, 28943 Fuenlabrada, Madrid Spain; 3 Faculty of Sport Science, University of Extremadura, Extremadura, Spain

**Keywords:** Rehabilitation, Randomized controlled trials

## Abstract

The objective of the present manuscript was to evaluate the effects of 24-weeks exergame intervention on the heart rate variability (HRV) of women with fibromyalgia. First, 56 women with fibromyalgia were assessed for eligibility. A total of 55 women fulfilled the inclusion criteria and participated in this single-blinded, randomized controlled trial. A 24-weeks of exergames were completed by the exercise group in the university facilities. It was focused on the mobility, postural control, upper and lower limbs coordination, aerobic fitness and strength. A total of 120 min per week, divided into two sessions of 60 min, was completed. A short-term 5 min record at rest was used to assess the HRV. Time (SDNN and RMSSD) and non-linear indexes (Higuchi´s Fractal Dimension, SD1, SD2, ln stress score, and SD1/SD2) of HRV were extracted. Fifty participants (achieving an 89.28% of adherence), recruited from the local fibromyalgia association completed the study. They were randomly divided into an exercise (age = 54.04[8.45]) and a control group (52.72[9.98]). Significant interaction (group*time) effects in SDNN, ln stress score, SD2, and SD1/SD2 ratio were found. The EG showed an increase of SDNN and a decreased ln stress score and SD2. The CG showed an increased ln stress score, SD1/SD2. In conclusion, 24-weeks of exergame intervention based on the tool VirtualEx-FM improved the autonomic control in patients with fibromyalgia. However, significant effects on Higuchi´s fractal dimension were not found. This is the first study using exergame as a therapy in women with fibromyalgia which has led to an improvement the autonomic balance in these patients.

## Introduction

Fibromyalgia is a chronic syndrome characterized by widespread pain. It is frequently accompanied by other symptoms, such as fatigue, stiffness or sleep disturbance^1^. In addition, previous studies have suggested an autonomic nervous system dysfunction (dysautonomia) in patients with FM^2–6^. Dysautonomia is characterized by persistent autonomic nervous system hyperactivity at rest as well as hyporeactivity during stressful situations^7^. Therefore, the dysautonomia might explain some of the multisystem features of FM^8^.

The heart rate variability (HRV) (beat-to-beat variation in the R-R interval) is a reproducible and non-invasive measure of the autonomic nervous system function^9^. It provides information about the autonomic modulation (balance between the parasympathetic and the sympathetic nervous system)^10^. It is a relevant measure since low HRV values are associated with an increased risk of death from several causes^11^. In patients with FM, HRV has been used to assess the autonomic dysfunction^2,3,5,6^. Moreover, non-linear methods have been introduced in the study of HRV. Whereas in healthy circumstances it has been observed a presence of power-law fractal organization, a degradation of the fractal scaling has been observed in pathologic conditions^12^. In this regard, women with fibromyalgia exhibits decreased nonlinearity and stronger anticorrelations in heart period fluctuations^13^.

A systematic review with synthesis of best evidence pointed out that one of the non-pharmacological therapies with the highest level of evidence to manage fibromyalgia symptoms is physical exercise^14^. Physical exercise has shown to increase HRV in patients with fibromyalgia in aerobic exercise, resistance training or hydrotherapy interventions^15–17^. However, this may be controversial since some studies have reported no changes in HRV after exercise intervention programs^18–20^.

Interventions based on virtual reality (VR) have emerged as a therapy in different populations^21–23^. Exergames are a non-immersive variation of VR which involve physical exercise^24^. In this regard, a recent systematic review and meta-analysis indicated that musculoskeletal pain was reduced after exergame interventions, especially in patients suffering from chronic pain. In addition, in patients with fibromyalgia, an 8-week exergame intervention improved the mobility skills^25^ and the quality of life and pain^26^.

However, to the best of our knowledge, there is no study evaluating the effects of exergames on HRV in patients with fibromyalgia. Therefore, the aim of the present study was to evaluate the effects of 24-weeks exergame-based intervention on HRV in patients with fibromyalgia. Based on the previously cited scientific studies, we hypothesized that participants would increase their HRV after the exergame-based intervention. This would reduce the persistent autonomic nervous system hyperactivity in women with fibromyalgia.

## Method

### Trial design

This study was conceived as a single-blinded, randomized controlled trial. Participants were randomly allocated into two groups: exercise group (EG) and control group (CG). All the procedures were approved by the University of Extremadura research ethics committee (approval number: 62/2017) and performed in accordance with relevant guidelines and regulations. The trial was prospectively registered at the International Standard Randomised Controlled Trial Number Registry (ISRCTN65034180, date: 07/12/2017). The protocol is available on the following website: 10.1186/ISRCTN65034180.

However, some changes were accomplished. First, sample size was increased in order to obtain a greater statistical power. Second, the intervention was performed in the university facilities instead of in the local fibromyalgia association facilities.

Three articles focused on the primary outcomes of the trial have been recently published^27–29^. Nevertheless, the hypothesis in the present study is entirely novel (improvements in HRV after an exergame intervention) and significantly differs from the other articles. Furthermore, this article involves a different research, with specific research professionals and audience, enabling us to deeply examine the findings in the autonomic modulation of women with fibromyalgia.

### Participants

The intervention was carried out in the University facilities (Faculty of Sport Science, Cáceres, Spain) from January 2018 to June 2018.

The fibromyalgia impact questionnaire was employed in order to estimate the sample size^30^. Taking into account that a 14% reduction is contemplated as clinically relevant^31^ and also that data from a previous research, the mean was expected to be 70.5 (11.8)^32^. Therefore, 26 participants per group were estimated in order to detect differences (α value 0.05 and 85% of power) of 14% in the fibromyalgia impact questionnaire.

Finally, a total of 56 women with fibromyalgia, from a fibromyalgia association were recruited until Dec 31, 2017. Participant inclusion criteria were:

- Be a woman between 30 and 75 years-old,
- Be able to communicate with the researchers and clinicians,
- Have read, accepted and signed the written informed consent,
- Be diagnosed with fibromyalgia by a rheumatologist according to the criteria of the American College of Rheumatology^1^.

In addition, participants were excluded if they:

- Modify their usual care therapies during the 24-week intervention,
- Have a condition that may make the physical exercise contraindicated according to a doctor, such as chronic or acute infectious diseases or renal, cardiac, pulmonary or hepatic failure.
- Be pregnant.

Participants were randomly allocated into the two groups (EG and CG) by one researcher using random numbers (EG and CG). The researcher who allocated the participants into the two groups did not take part in the acquisition or data analysis. A researcher who was blinded to the grouping allocation developed the evaluations. Nevertheless, participants were not blinded since they have to sign written informed consent about exergame intervention.

### Interventions

EG consisted of 24-weeks of exergame-based intervention (two sessions of one hour per week) in groups of two or three participants. By contrast, the CG continued with their usual daily life. The intervention and evaluations were carried out in the university facilities. The intervention was delivered as planned.

The exercise intervention was based on the VirtualEx-FM exergame program. This is a tool specifically created by our research group which aimed to improve the ability to activities of daily living through the improvements in strength, postural control, aerobic fitness, mobility, and coordination of the upper and lower limbs^26^ in patients with fibromyalgia. Collado-Mateo, *et al*.^26^ and Collado-Mateo, *et al*.^25^ described in their study how this tool fulfilled with eight key points to consider the VirtualEx-FM as a virtual reality rehabilitation therapy^33^.

A typical session of the exergame intervention contained:

1. A warm-up where participants have to do joints movements guided by a video of a kinesiologist;
2. Aerobic component following the teacher´s dance steps,
3. Coordination and postural control games where participants have to reach an apple that appears and disappears in different locations. The application indicated the body segment participants have to use. Moreover, it can be manually controlled by the kinesiologist; and,
4. Walking training where the participant must comprise a virtual trail of footprints. The interface allows the selection of different kind of steps such as: tiptoe, raised heels or knees, heel walking or normal. More details of the VirtualEx-FM are available in Collado-Mateo, *et al*.^26^ and Collado-Mateo, *et al*.^25^.

### Outcomes

A short-term HRV register (5-minutes in sitting position) was recorded pre and post intervention. The heart rate monitor Polar RS800CX (Finland) was used^34^. Recommendations of the *Task Force of the European Society of Cardiology and the North American Society of Pacing and Electrophysiology*^35^ as well as instructions derived from previous studies were followed^36,37^. The Kubios HRV software (v. 2.1)^38^ was employed to extract time domain non-linear measures.

In order to correct artifacts, a medium filter was applied identifying all beat to beat intervals (RR) which were longer/shorter than 0.25 seconds (compared to the local average)^39^. A cubic spline interpolation replaced the artifacts. Low-frequency baseline trend components were removed using the smoothness prior method with a Lambda value of 500^40^.

### Time-domain

SDNN: the standard deviation of all normal to normal RR intervals. Both sympathetic and parasympathetic systems contribute to SDNN. However, in short-term resting conditions the primary source of variations is parasympathetically-mediated by the respiratory sinus arrhythmia^9^.

RMSSD: the root mean square of successive differences between RR intervals. Since this measure reflects the beat-to-beat variance, it is used to estimate the vagally mediated changes in HRV^9,41^.

### Non-linear measures

SD1, SD2 and SD1/SD2 ratio: the standard deviation, of points perpendicular to the axis of line-of-identity in the Poincaré plot (SD1), the dispersion, standard deviation, of points along the axis of line-of-identity in the Poincaré plot (SD2) and the ratio between SD1 and SD2 (SD1/SD2 ratio) were calculated. The SD1 reflects the short-term HRV and it is identical to RMSSD measure^42^. The SD2 measures both, short and long-term HRV and correlates with LF^43,44^. The ratio SD1/SD2 is traditionally employed to measure the autonomic balance, correlating with the LF/HF ratio^45,46^.

ln stress score: the natural logarithm of the 1000*1/SD2. This index was created to provide a directly proportional value of the sympathetic activity^47^.

Higuchi Fractal dimension (HFD): HFD^48^ was calculated using the algorithm described by Khoa, *et al*.^49^ with the MATLAB R2016b software (The Mathworks Inc., Natick, MA, United States; Academic License, IBBE PAS). Firstly, as proposed by Kantelhardt, *et al*.^50^ the original time series *y*(i) were integrated from its mean, ȳ, for every time point *i*, as follow:

$$y(k)=\mathop{\sum }\limits_{i=1}^{k}[y(i)-\bar{{\rm{y}}}]\,{\rm{for}}\,k=1,\mathrm{..}.,N$$

where *N* is the length of the time series.

Subsequently, to obtain the fractal dimension, the N-length data series were split to a k-series set. This process calculates the length of the curve of each series in k-series set. The length for total curve was calculated using average. This gives L(k) for a k which varied from 1 to Kmax. Kmax was calculated as: floor(length(data)/2). The fractal dimension was calculated over the entire 5-min epoch using all data points.

### Statistical analysis

The SPSS statistical package (version 20.0; SPSS, Inc., Chicago, Ill.) was used to analyze the data.

Parametric tests were conducted based on the results of Shapiro-Wilk and Kolmogorov-Smirnov tests. Moreover, surrogate data were generated in order to test if the original data were derived from a stationary linear stochastic process with Gaussian inputs. This process consists of generating a surrogate data set with the same linear properties of the original RR data. If the comparison between surrogate data and RR time series is significantly different, the null hypothesis is rejected and nonlinearity assumed^51,52^. Surrogate data analysis was performed with the RHRV^53^, an open-source package for the statistical environment R (see: http://rhrv.r-forge.r-project.org/index.html), following the steps of Martínez, *et al*.^53^. In addition, Chi-squared test was used to evaluate differences between control and exergame in the nonlinearity assumption.

Repeated Measures ANOVA with Bonferroni correction for multiple comparisons to avoid the increase of type I error was conducted to explore the effects of the exergame intervention. Within groups comparisons, between the pre and the post-tests, were performed by the T-tests for paired samples. Cohen´s D effect size was reported for each statistical test^54^.

## Results

**Figure 1:**
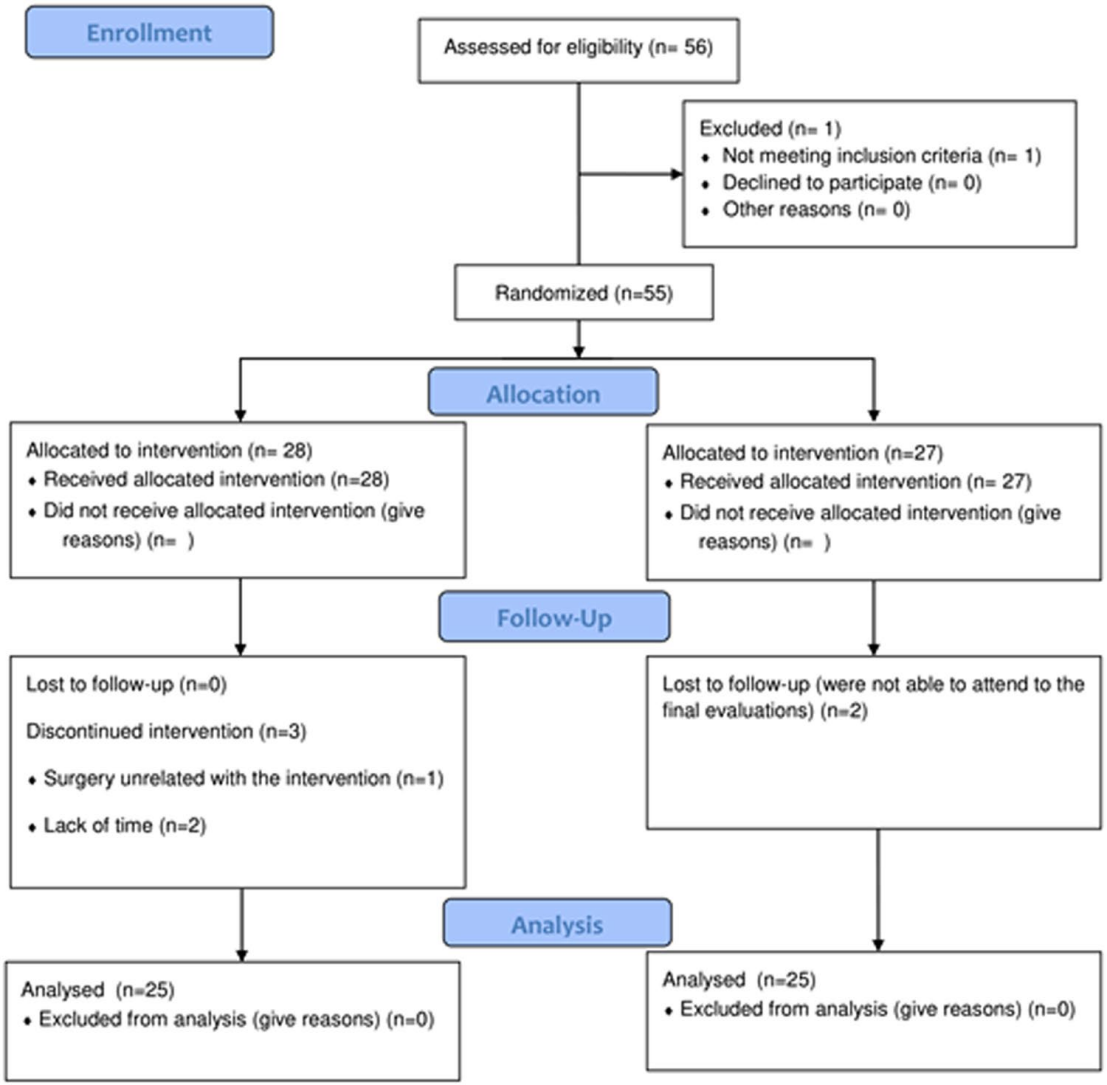
Flow diagram of the participants.

The flow diagram of the participants is depicted in Fig. 1. A total of 56 patients with fibromyalgia were screened for eligibility. One woman did not meet the inclusion criteria, so she was excluded. Lastly, 55 women were randomized into two groups: EG and CG. Regarding the compliance with the treatment, 50 women finished the intervention program (EG: n = 25; age = 54.04 [8.45] and CG: n = 25; age = 52.72 [9.98]). Three women allocated in the EG and two in the CG were lost to follow-up. In the EG the causes were a surgery unrelated to the exercise intervention (n = 1) and the lack of time (n = 2). In the CG two women were not able to attend the final evaluations. The intervention was considered as completed when the participant attended a minimum of 75%. Thus, considering this data the final adherence was 89.28%. No side effects derived from the intervention were detected.

The main characteristics of the participants at baseline are summarized in Table 1. At baseline, no significant differences were reported in age nor the main outcomes (HRV indexes).

**Table 1:** Demographic data and differences between groups at baseline.

Variable	Exercise Group Mean (SD)	Control Group Mean (SD)	Value of Contrast	p-value^a^
Sample size	28	27		
Age (years)	54.04 (9.96)	53.41 (9.92)	0.234	0.816
SDNN (ms)	22.98 (8.12)	26.61 (10.28)	−1.455	0.152
RMMSD (ms)	21.18 (10.51)	22.97 (12.88)	−0.565	0.574
ln Stress Score	3.61 (0.33)	3.47 (0.44)	1.261	0.213
SD1 (ms)	15.01 (7.44)	16.26 (9.12)	−0.561	0.577
SD2 (ms)	28.55 (9.49)	33.59 (12.39)	−1.697	0.096
SD1/SD2	0.52 (0.15)	0.47 (0.16)	1.251	0.216
HFD	1.33 (0.12)	1.37 (0.12)	−0.915	0.365

^a^p-values of t-test for independent sample to compare differences between groups at baseline.

SDNN: standard deviation of all normal to normal RR intervals; ln stress score: natural logarithm of the 1000*1/SD2; SD1: standard deviation of points perpendicular to the axis of line-of-identity in the Poincaré plot; SD2: standard deviation, of points along the axis of line-of-identity in the Poincaré plot; SD1/SD2: ratio between SD1 and SD2; HFD: Higuchi Fractal Dimension.

Surrogate data was calculated for each participant in the pre and post RR time series. Nonlinearity, in the pre-test, is assumed in 24 participants of the exercise (n = 12, 48%) and control (n = 12, 48%) groups. Similar results were found in the post-test where nonlinearity is assumed in fourteen (32%) and eleven (40%) participants of the exergame and control group respectively. Chi-square test did not show differences between control and exergame groups in the nonlinearity assumption in both pre and post-tests (see Table 2). The efficacy analysis of the intervention is reported in Table 3. The repeated measures ANOVA showed significant interaction effects (time*group) in SDNN, ln stress score, SD2, and SD1/SD2 ratio. In this regard, in the EG, T-test for paired sample showed an increase of SDNN and a decrease of ln stress score and SD2. In the CG, T-test for paired sample showed an increase of ln stress score (see Table 3). No significant effects were obtained in the remaining HRV indexes (see Table 3).

**Table 2:** Surrogate data test analysis of the RR time series.

		Non linearity assumed n (%)	
		Pre	Post
Surrogate data test	Exercise	12 (48%)	14 (56%)
	Control	12 (48%)	11 (44%)
p-value		1.000	0.720

^a^p-values obtained from chi-square test.

**Table 3:** Efficacy analysis of exergame intervention in the HRV indexes in patients with fibromyalgia.

Heart rate variability indexes		Pre	Post	Within Group Comparison			Between Group Comparison		
				Value of the contrast	P-value	Effect Size	F	P-value^a^	Effect Size
SDNN (ms)	Exercise	23.59 (8.38)	28.37 (13.83)	−2.454	0.022	−0.368	6.262	0.016	0.721
	Control	25.64 (10.06)	20.97 (12.28)	1.443	0.162	0.415			
RMSSD (ms)	Exercise	21.56 (11.06)	24.28 (16.97)	−0.806	0.428	−0.333	0.592	0.446	0.220
	Control	21.07 (11.11)	20.14 (14.36)	0.280	0.782	0.073			
ln Stress Score	Exercise	3.58 (0.33)	3.42 (0.42)	3.119	0.005	0.414	7.739	0.008	0.804
	Control	3.50 (0.45)	3.83 (0.66)	−1.950	0.063	−0.587			
SD1 (ms)	Exercise	15.27 (7.83)	17.20 (12.01)	−0.807	0.428	−0.187	0.593	0.445	0.220
	Control	14.92 (7.87)	14.26 (10.17)	0.279	0.783	0.072			
SD2 (ms)	Exercise	29.37 (9.73)	35.86 (16.36)	−3.124	0.005	−0.395	9.024	0.004	0.866
	Control	32.82 (12.55)	25.77 (14.49)	1.763	0.091	0.520			
SD1/SD2	Exercise	0.52 (0.16)	0.46 (0.17)	1.224	0.233	0.351	6.897	0.012	0.759
	Control	0.44 (0.13)	0.54 (0.14)	−2.748	0.011	−0.687			
HFD	Exercise	1.33 (0.12)	1.34 (0.15)	−0.132	0.896	−0.034	1.010	0.320	0.293
	Control	1.37 (0.12)	1.32 (0.11)	1.250	0.224	−0.320			

## Discussion

The aim of the present study was to evaluate the effects of a 24-weeks exergame intervention on the HRV in patients with fibromyalgia. Exergames have been used previously to improve both physical function and quality of life in women with fibromyalgia after 8-week^25,26^ and 24-week^27,28^ interventions. However, this is, to our knowledge, the first study to report a significant effect of an exergame-based intervention on the HRV of patients with fibromyalgia.

Fibromyalgia patients showed an abnormal autonomic modulation compared with healthy controls^6,7^. Importantly, our results showed that exergame promoted a change in autonomic modulation after the intervention by a significant increase in SDNN and SD2 and a significant decrease in ln Stress Score and SD1/SD2. Similar results have been observed in previous studies. In this regard, in a 24-weeks of aerobic training, fibromyalgia patients improved HRV measures such as lnLF and lnLF/HF^15^. In the same line, Zamuner, *et al*.^17^ found that 16-weeks of hydrotherapy intervention (based on aerobic, resistance and stretching exercises) improved the HRV when compared with healthy controls (lnHF measure). Furthermore, 16-weeks of resistance training^16^ showed changes in the HRV (total power, lnHF and RMSSD measures), when compared with healthy controls. Similar effects were found after 12-week Tai Chi intervention (significant effects in lnLF/HF ratio, lnHF and lnLF)^55^. However, there are also previous studies that did not find significant changes on HRV^18–20^ after 16 weeks of strengthening^18^, 8 weeks of resistance exercise^19^, 12 weeks of resistance training respectively^20^ and 12 weeks of moderate intensity spinning workouts^56^. Our results support the idea of vagal tone recovery due to exercise interventions. Furthermore, considering both our results and the previous research, it can be hypothesized that interventions longer than 4 months and comprising resistance or aerobic exercises are necessary to improve HRV. This hypothesis is also supported by previous studies in other populations^57,58^.

The non-linear measures seem to be more sensitive to autonomic modulation changes induced by exercise intervention than time measures since significant effects were found in the ln stress score, SD2 and the SD1/SD2 ratio. One of these indices, the ln stress score, was originally developed to control the autonomic balance in elite soccer players^47^. However, our results indicate that this index could be an interesting tool to measure the sympathetic activity, also in a different population with abnormal autonomic modulation such as fibromyalgia. This index is also derived from the Poincaré plot, i.e. ln stress score = ln (1000*1/SD2). However, significant effects of exergames in the chaotic behavior of HRV measured by the HFD have been not found. The HFD is an appropriate method for analyzing the fractal dimension of biomedical signals^59^ in order to quantify self-similarity and complexity of the signal, providing more accurate estimation of fractal dimension than other proposed methods^59,60^. It has been applied to brain^61^, cardiac^62^ or even center of pressure signals^63^. In this regard, a previous study with healthy and diabetic populations used the HFD to determine the effects of Percutaneous Auricular Vagus Nerve Stimulation on the Autonomic Nervous System^64^. Moreover, a decrease in fractal dimension has been observed under stress situations^65^. This is in line with the acute decrease of HFD after physical exercise^66^. However, further studies are needed to completely understand the interrelation of HFD with Autonomic Nervous System measures.

These results are relevant since lower values of HRV are related to high risk of mortality^11^. In addition, a previous study reported that improvements in HRV non-linear indexes were related to improvement in pain, quality of life and impact of the disease^17^. In this regard, Martinez-Lavin, *et al*.^67^ proposed that the sympathetic hyperactivity could be related to insomnia, anxiety and chronic pain suffered by patients with fibromyalgia. This could be explained by hyperactivity of the sympathetic nervous systems to stress, resulting of the down-regulation and desensitization of adrenergic receptors.

Nonlinear dynamics are involved in the creation of HRV due to different hemodynamic electrophysiological and autonomic/central regulations^68,69^. In this regard, surrogate data analysis has emerged as one of the most popular test to determine the presence of nonlinear dynamics in the RR time series^69^, assessing the capability of the different nonlinear indexes to detect nonlinearity in HRV data^70–73^. Our results showed that nonlinearity is assumed in 49% of the total RR data series (including pre and post-tests). Therefore, results from non-linear variables, such as HFD, ln Stress Score, SD1, SD2 and SD1/SD2, must be taken with caution. Nevertheless, differences between control and exergame groups in the nonlinearity assumption were not found.

### Study limitations

This study has some limitations that should be mentioned. First, a third group that performed exercise training intervention not based in VR should be recommended to isolate the effects of an exergame intervention. This fact makes that the effects of VR intervention should be taken with caution. Second, due to the relatively small sample probably only great differences have reached the statistical significance level. Third, in the present study only participated women, so we cannot generalize the results to men with fibromyalgia. Fourth, regarding non-linear analysis surrogate data analysis indicated that in 49% of RR data series intervals nonlinearity is assumed. Therefore, results from non-linear variables should be taken with caution.

## Conclusions

To summarize, 24-weeks of exergame intervention based on the tool VirtualEx-FM improved the autonomic control in patients with fibromyalgia. However, effects of exergames chaotic behavior of HRV measured through the HFD were not found. This is the first study using exergames as a therapy in women with fibromyalgia achieving improvements in the autonomic control.
